# Study on the Effect of “3D-rGO” Buffer Layer on the Microstructure and Properties of SiO_2f_/SiO_2_ and TC4 Brazed Joint

**DOI:** 10.3390/ma17061394

**Published:** 2024-03-19

**Authors:** Peng Liu, Qiang Ma, Yongwei Chen, Shujin Chen, Jie Zhu, Peng He, Xiaojiang Chen, Xiao Jin, Bin Zheng

**Affiliations:** 1 Key Laboratory of Advanced Welding Technology of Jiangsu Province, Jiangsu University of Science and Technology, Zhenjiang 212003, China; 2 State Key Laboratory of Advanced Welding and Joining, Harbin Institute of Technology, Harbin 150001, China; 3 Zhejiang Seleno Science and Technology Co., Ltd., Jinhua 321016, China

**Keywords:** SiO_2f_/SiO_2_ composite, wettability, residual stress, “3D-rGO” buffer layer, shear strength

## Abstract

Brazing a SiO_2f_/SiO_2_ composite with metals is often faced with two problems: poor wettability with the brazing alloy and high residual stress in the joint. To overcome these problems, we report a combined method of selective etching and depositing reduced graphene oxide (rGO) on the surface of a SiO_2f_/SiO_2_ composite (3D-rGO-SiO_2f_/SiO_2_) to assist brazing with TC4. After the combined treatment, a “3D-rGO” buffer layer formed on the surface layer of the SiO_2f_/SiO_2_, and the contact angle was reduced from 130° to 38°, which meant the wettability of active brazing alloy on the surface of SiO_2f_/SiO_2_ was obviously improved. In addition, the “3D-rGO” buffer layer contributed to fully integrating the brazing alloy and SiO_2f_/SiO_2_; then, the infiltration of the brazing alloy into the surface layer of the SiO_2f_/SiO_2_ was enhanced and formed the reduced graphene oxide with a pinning structure in the three dimensional (“3D-pinning-rGO”) structure. Moreover, the joining area of the brazing alloy and SiO_2f_/SiO_2_ was expanded and the mismatch degree between the SiO_2f_/SiO_2_ and TC4 was reduced, which was achieved by the “3D-pinning-rGO” structure. Furthermore, the concentration of the residual stress in the SiO_2f_/SiO_2_-TC4 joints transferred from the SiO_2f_/SiO_2_ to the braided quartz fibers, and the residual stress reduced from 142 MPa to 85 MPa. Furthermore, the 3D-pinning-rGO layer facilitated the transfer of heat between the substrates during the brazing process. Finally, the shear strength of the SiO_2f_/SiO_2_-TC4 joints increased from 12.5 MPa to 43.7 MPa by the selective etching and depositing rGO method.

## 1. Introduction

SiO_2f_/SiO_2_ composite is braided by quartz fibers in three dimensions and fused silica is treated as a filler [1]. Because of the low mass density, good transmissivity and decent thermomechanical properties, a SiO_2f_/SiO_2_ composite is used in the aerospace industry [2]. However, due to its poor machinability and inherent brittleness, SiO_2f_/SiO_2_ is difficult to use alone for fabricating complex structural parts. In order to expand the application scope, SiO_2f_/SiO_2_ is usually joined with metals. With a light mass, high hardness and good elevated temperature properties, TC4 (Ti6Al4V) is an ideal material for the key equipment of aircraft and missiles [3]. As a result, it is very important to realize the high-quality joining of SiO_2f_/SiO_2_ and TC4. Many studies revealed that brazing is one of the most suitable methods to join ceramic and ceramic matrix composite with metals [4,5,6,7,8,9,10]. However, when brazing SiO_2f_/SiO_2_ composite and TC4, two problems exist. First, the wettability of an active brazing alloy on the surface of SiO_2f_/SiO_2_ is poor due to no metallurgical bonding between the active brazing and SiO_2f_/SiO_2_. Second, high residual stress is produced in the joint due to the large mismatching degree of the coefficient of thermal expansion (CTE) [11,12,13,14,15]. By resolving the above two problems, a high-quality SiO_2f_/SiO_2_-TC4 can be achieved.

Recently, many researchers focused on the wettability of SiO_2f_/SiO_2_. Qi et al. [16] reported on the plasma treatment of SiO_2f_/SiO_2_ by PECVD for their assisted brazing with Nb. The results revealed that after the treatment, a carbon film and Si-C bonding formed on the surface layer of SiO_2f_/SiO_2_, which was easily wetted by an active brazing alloy, and the contact angle of AgCuTi on C-SiO_2f_/SiO_2_ was reduced from 131° to 27°. Sun et al. [17] modified the surface of SiO_2f_/SiO_2_ with active few-layer graphene (FLG) to improve the wettability of a AgCuTi alloy on it. It showed that the synthesized FLGs processed high chemical reactivity and AgCuTi alloy preferentially reacted with them, and thus, the contact angle of AgCuTi alloy on the surface of SiO_2f_/SiO_2_ was decreased from 123.8° to 50.7°. In Lin et al. [18], graphene oxide (rGO) was diluted and coated on the surface of SiO_2f_/SiO_2_, and the sample was heated at 200 °C for 30 min. They explained that rGO had more defective sites to react with the Ti element and improved the wettability of SiO_2f_/SiO_2_. Then, the contact angle decreased from 131° to 42.8°. Then, it can be inferred that the active carbon layer contributed to promoting the reaction between the brazing alloy and the carbon layer, and the wettability of the brazing alloy on the surface of SiO_2f_/SiO_2_ was improved. However, due to the small addition of a carbon layer, the residual stress in the joint was limitedly relieved. Therefore, many researchers were also interested in methods to release residual stress in the joint. Liu et al. [19] directly joined copper and AlN ceramic without a brazing alloy through laser-induced metallization of an AlN ceramic. They found that the micro-groove structures fabricated by laser irradiation significantly increased the jointing area of the joints and played the role of mechanical interlocking, which was conducive to improving the shear strength. Furthermore, the residual stress was difficult to measure by experimental methods [20,21,22,23,24]. Therefore, many researchers researched the residual stress in the joint with the help of finite element simulations (FEMs) [25,26,27,28,29]. Ma et al. [30] regulated the surface structure of SiO_2f_/SiO_2_ by selective etching to assist with brazing with Nb. They explained that during brazing, the brazing alloy infiltrates SiO_2f_/SiO_2_ and forms a “3D-pinning” structure in the joint. In addition, the FEM results show that when the depth of the structure was 100 μm, the distribution of residual stress in the joint was optimal. Then, it can be inferred that the “3D-pinning” structure with suitable dimensions was able to effectively release the residual stress in the joint.

In this study, a simple and efficient “3D-rGO” buffer layer was first modified by the combined method of selective etching and deposited treatment on the surface of SiO_2f_/SiO_2_ composite for assisting in brazing with TC4. This investigation emphasized the effect of “3D-rGO” on the wettability of active brazing alloy on the surface of the SiO_2f_/SiO_2_ composite. Then, the parameters of the “3D-rGO” buffer layer were further optimized to achieve a reliable SiO_2f_/SiO_2_-TC4 joint. The effects of the concentration of the 3D-rGO on the wettability of the AgCuTi alloy on the surface of the SiO_2f_/SiO_2_ composite and the distribution of the residual stress in the joints were comprehensively analyzed. And the relationship between interfacial structure and residual stress was explained by finite element simulations (FEMs). Furthermore, the strengthening mechanism of the SiO_2f_/SiO_2_-TC4 joint by the “3D-pinning-rGO” structure was revealed in detail.

## 2. Experimental Methodology

**Figure 1:**
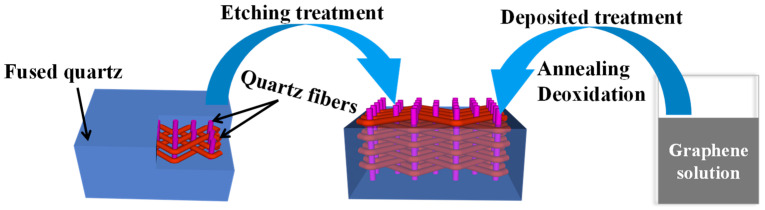
Schematic of surface treatment of SiO_2f_/SiO_2_.

The base materials were a SiO_2f_/SiO_2_ composite (China Aerospace and Technology Corporation, Beijing, China) and a commercial TC4 alloy. For the SiO_2f_/SiO_2_ composite, the structure was three-dimensional, and quartz fibers served as the braided body, with fused quartz as the filler. All the brazing surfaces were polished by SiC paper and ultrasonically cleaned in ethanol for 15 min. The substrates had two sets of sizes: (1) SiC (5 × 5 × 3 mm^3^) and TC4 (10 × 10 × 3 mm^3^) were used for the mechanical property measurements; (2) SiC (10 × 10 × 3 mm^3^) and TC4 (10 × 10 × 3 mm^3^) were used for the microstructure observations. The preparation procedure of the rGO@E-SiO_2f_/SiO_2_ is shown in Figure 1. For the etching treatment, 20 wt% HF acid solution was applied for 20 s on the surface of the SiO_2f_/SiO_2_ composite, and the composite was cleaned in ethanol for 5 min; then, E-SiO_2f_/SiO_2_ was obtained. For the deposited treatment, a drop of rGO solution (about 0.05 mL with concentrations of 0 mg/mL, 0.5 mg/mL, 1.0 mg/mL, 1.5 mg/mL and 2.0 mg/mL) was dripped on the surface of the E-SiO_2f_/SiO_2_, and then the samples were put in the oven at 200 °C for 60 min. Then, the deposited treatment was repeated 2–3 times, and rGO@E-SiO_2f_/SiO_2_ was obtained.

Wetting experiments were tested by the sessile drop method at 860 °C for 10 min in a high-vacuum system. First, AgCuTi brazing alloy foil, which was rolled into a roll (about 20 mg), was put on the surface of the rGO@E-SiO_2f_/SiO_2_. Second, the samples were placed in wetting equipment (OCA, DataPhysics Corporation, Hamburg, Germany) and heated to 860 °C for 10 min. Then, the contact angle (the wettability of AgCuTi brazing alloy on the surface of SiO_2f_/SiO_2_ composite) was measured by an imaging device in the wetting equipment. For the brazing experiments, the AgCuTi brazing alloy was placed between the rGO@E-SiO_2f_/SiO_2_ and TC4 with a sandwich structure; then, the assemblies were transferred into a furnace with a 2~4 × 10^−4^ Pa vacuum. During the brazing process, the brazing samples were heated to 860 °C at 10 °C/min, held for 10 min, then cooled down to room temperature at 5 °C/min.

The morphology and interfacial microstructure of the joints were observed by a scanning electron microscope (SEM) fitted with an energy dispersive spectrometer (EDS) and X-ray diffraction (XRD). The average shear strengths of the joints were examined by five shear samples using an Instron-1186 universal testing machine (Shengyangkejing, Shengyang, China).

## 3. Results and Discussion

### 3.1. Wettability Enhancement

**Figure 2:**
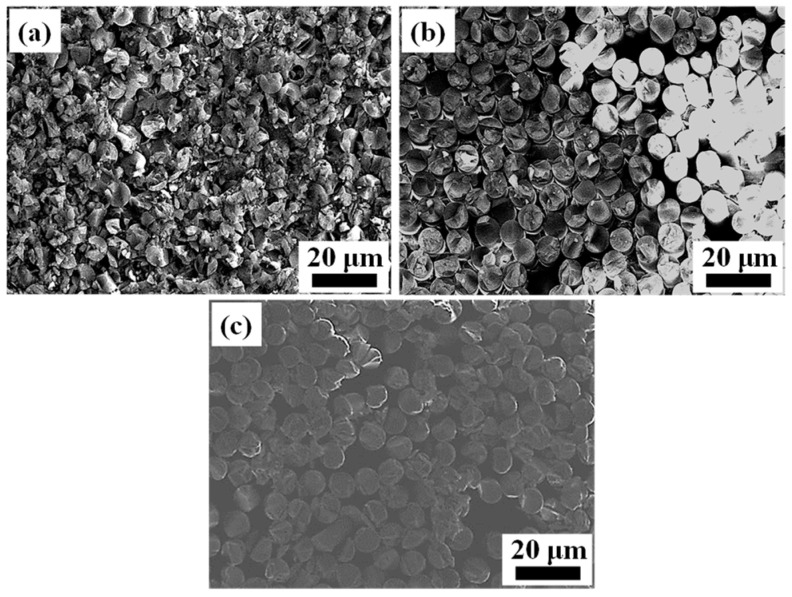
The surface microstructure of (**a**) original SiO_2f_/SiO_2_, (**b**) E-SiO_2f_/SiO_2_ and (**c**) rGO@E-SiO_2f_/SiO_2_.

Figure 2a–c show the cross-section of the morphology of the original SiO_2f_/SiO_2_, E-SiO_2f_/SiO_2_ and rGO@E-SiO_2f_/SiO_2_, respectively. From Figure 2a, it can be seen that the SiO_2f_/SiO_2_ body mainly consisted of numerous quartz fibers braided in three dimensions, and fused quartz was treated as the filler. After the selective etching treatment, the surface layer of the SiO_2f_/SiO_2_ only consisted of numerous quartz fibers; moreover, signs of corrosion could be found on the surface of quartz fibers, as shown in Figure 2b. This means that the HF solution was able to etch the quartz fibers and fused quartz. Compared with the SiO_2f_/SiO_2_, an extremely thin rGO layer adhered to the surface of the rGO@E-SiO_2f_/SiO_2_ and the rGO filled the gap of fused quartz on the surface layer of the SiO_2f_/SiO_2_, as shown in Figure 2c. Then, it can be inferred that a “3D-rGO” buffer layer was achieved by the combined method of selective etching and deposited treatment.

**Figure 3:**
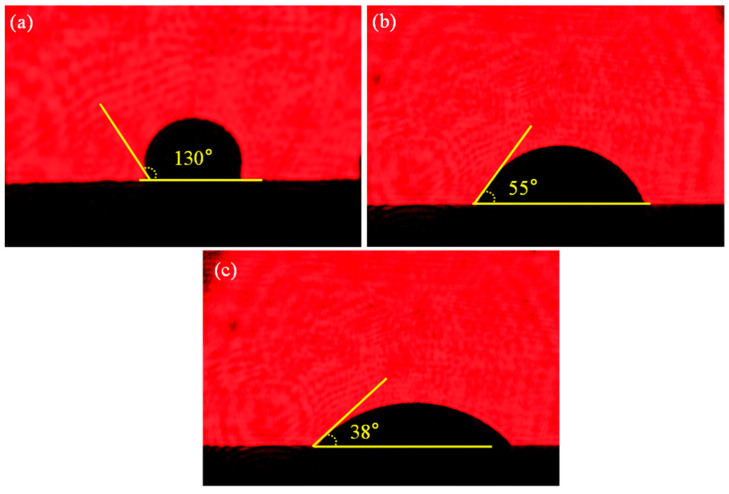
The contact angle of AgCuTi alloy on the surface of (**a**) original SiO_2f_/SiO_2_, (**b**) E-SiO_2f_/SiO_2_ and (**c**) rGO@E-SiO_2f_/SiO_2_.

In order to compare the wettability of SiO_2f_/SiO_2_, E-SiO_2f_/SiO_2_ and rGO@E-SiO_2f_/SiO_2_ with AgCuTi alloy, wetting experiments were carried out at 860 °C for 10 min. It can be found from Figure 3a that the contact angle of AgCuTi on the surface of SiO_2f_/SiO_2_ was 130°, meaning the wettability of the AgCuTi on the surface of the SiO_2f_/SiO_2_ was poor. As for E-SiO_2f_/SiO_2_, the contact angle was reduced to 55°, which meant the wettability of AgCuTi on the surface of SiO_2f_/SiO_2_ was improved (see Figure 3b). This may have been because the fused silicon, which caused the poor wettability of the AgCuTi alloy on the surface of SiO_2f_/SiO_2_, was consumed, and the wettability of the AgCuTi on the surface of quartz fibers was good. Therefore, after the selective etching treatment, the quartz fibers were conducive to filling in the blanks of the fused quartz by the AgCuTi brazing alloy in the surface layer of the SiO_2f_/SiO_2_; then, the wettability of the AgCuTi on the surface of the SiO_2f_/SiO_2_ was improved. As seen in Figure 3c, the contact angle of the AgCuTi alloy on the rGO@E-SiO_2f_/SiO_2_ was further reduced to 38°. This may have been because the reaction between the element Ti and the element C promoted the spreading and wetting of the AgCuTi alloy on the surface of the rGO@E-SiO_2f_/SiO_2_. Therefore, it can be inferred that a thin “3D-rGO” buffer layer formed on the surface layer of the SiO_2f_/SiO_2_ by the combined selective etching treatment of the coating, and the wettability of the SiO_2f_/SiO_2_ was significantly improved by the buffer layer.

**Figure 4:**
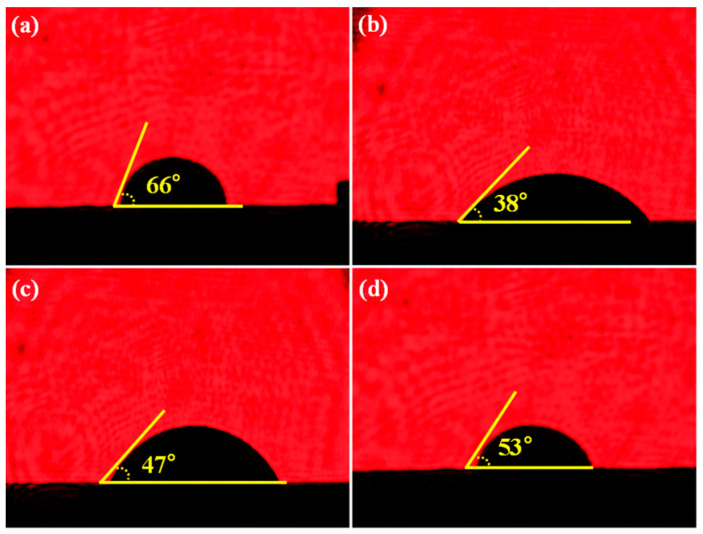
The contact angle of AgCuTi alloy on the surfaces of (**a**) 0.5 mg/mL rGO@E-SiO_2f_/SiO_2_, (**b**) 1.0 mg/mL rGO@E-SiO_2f_/SiO_2_, (**c**) 1.5 mg/mL rGO@E-SiO_2f_/SiO_2_ and (**d**) 2.0 mg/mL rGO@E-SiO_2f_/SiO_2_.

Furthermore, in order to analyze the effect of the concentration of rGO on the wettability of the AgCuTi alloy on the surface of the SiO_2f_/SiO_2_, the wetting experiments of the SiO_2f_/SiO_2_ with different “3D-rGO” buffer layers were conducted. The contact angle of the AgCuTi on the surface of the 0.5 mg/mL rGO@E-SiO_2f_/SiO_2_, 1.0 mg/mL rGO@E-SiO_2f_/SiO_2_, 1.5 mg/mL rGO@E-SiO_2f_/SiO_2_ and 2.0 mg/mL rGO@E-SiO_2f_/SiO_2_ are shown in Figure 4. From Figure 4a–d, it can be seen that the contact angle decreased from 66° to 38° as the concentration of rGO increased from 0.5 mg/mL to 1.0 mg/mL. Then, the contact angle increased to 53° as the concentration of rGO increased to 2.0 mg/mL. It is worth noting that as the concentration of rGO increased, the wettability of the AgCuTi alloy on the surface of the SiO_2f_/SiO_2_ improved first and then deteriorated. It can be inferred that as the concentration of rGO increased from 0.5 mg/mL to 1.0 mg/mL, the metallurgical bonding between the AgCuTi alloy and the SiO_2f_/SiO_2_ was enhanced and the wettability of the SiO_2f_/SiO_2_ was improved. Meanwhile, as the concentration of the rGO further increased to 2.0 mg/mL, the metallurgical bonding between the AgCuTi alloy and SiO_2f_/SiO_2_ was suppressed due to the fluidity of the AgCuTi alloy becoming bad and the wettability of the SiO_2f_/SiO_2_ becoming deteriorated. Therefore, it can be concluded that when the concentration of the rGO was 1.0 mg/mL, the wettability of the AgCuTi on the surface of the SiO_2f_/SiO_2_ composite was appropriate.

### 3.2. Microstructure Integration

**Figure 5:**
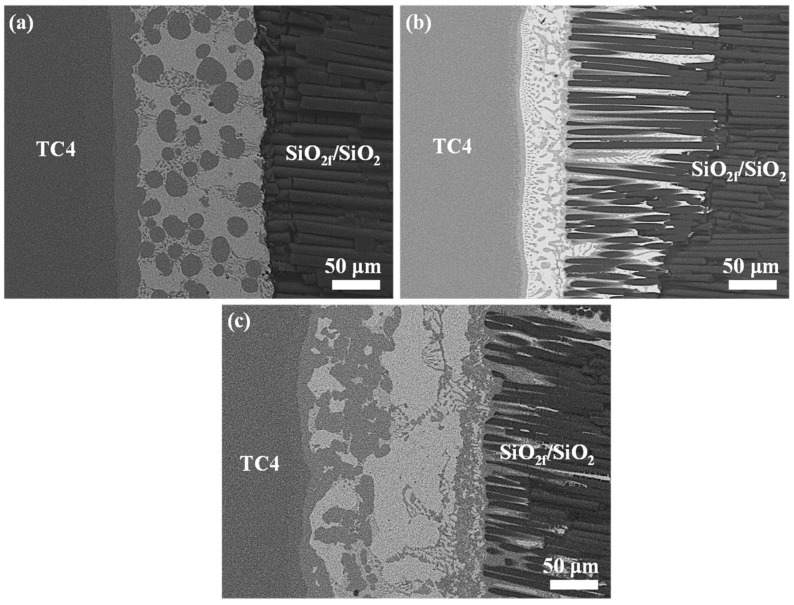
The microstructure of (**a**) SiO_2f_/SiO_2_-TC4 joint, (**b**) E-SiO_2f_/SiO_2_-TC4 joint and (**c**) rGO@E-SiO_2f_/SiO_2_-TC4 joint.

**Figure 6:**
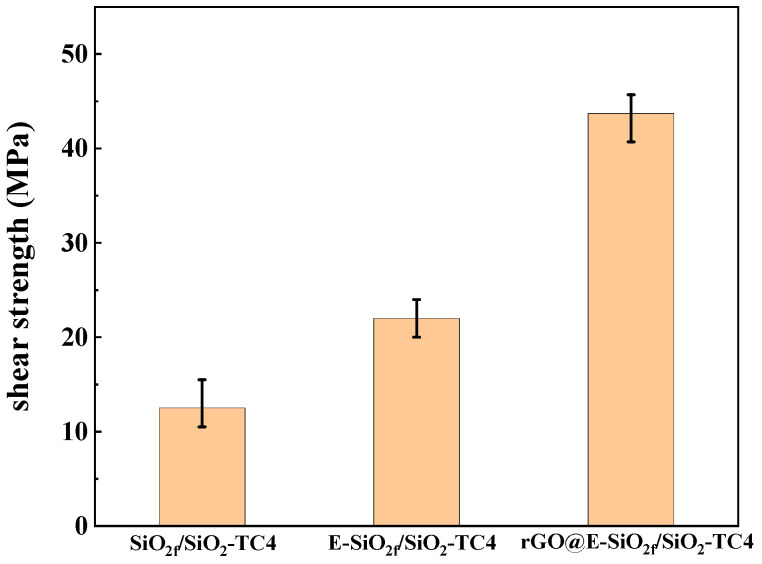
The shear strengths of the joints.

**Figure 7:**
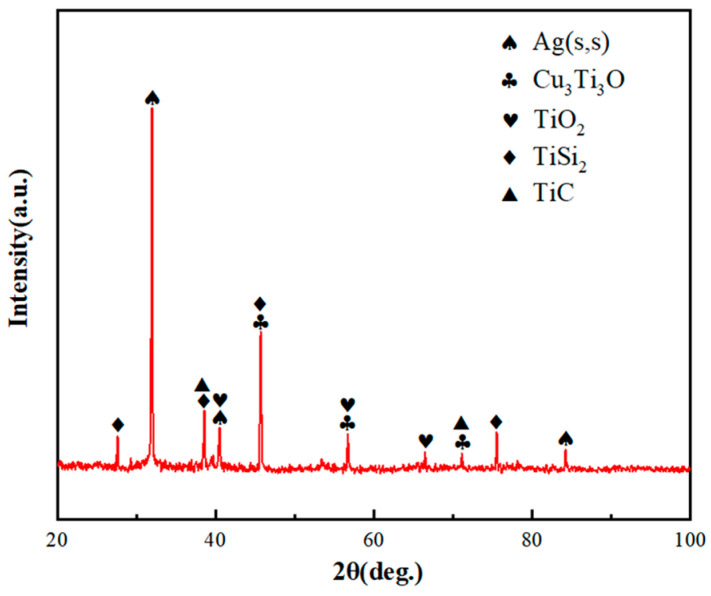
XRD analysis of buffer layer in rGO@E-SiO_2f_/SiO_2_-TC4 joint.

To find out the mechanism of the enhanced joints, a brazing experiment was carried out at 860 °C for 10 min. Typical microstructures of the SiO_2f_/SiO_2_-TC4 joint, E-SiO_2f_/SiO_2_-TC4 joint and rGO-E-SiO_2f_/SiO_2_-TC4 joint are shown in Figure 5. As for the SiO_2f_/SiO_2_-TC4 joints (see in Figure 5a), a distinct crack appeared at the brazing seam near the SiO_2f_/SiO_2_ side and the shear strength of the joints was only 12.5 MPa (see in Figure 6). This may have been because the wettability of the AgCuTi alloy on the surface of the SiO_2f_/SiO_2_ was poor and the residual stress produced by the large mismatch degree of the CTE between the SiO_2f_/SiO_2_ and the TC4 was high. After the SiO_2f_/SiO_2_ composite was treated by selective etching, a “3D-pinning” structure was presented in the microstructure of the E-SiO_2f_/SiO_2_-TC4 brazed joints. Cracks formed at the bottom of the “3D-pinning” structure and the shear strength of the joints reached 22 MPa (see Figure 5b and Figure 6). This may have been because, after the etching treatment, a “3D-pinning” structure, which mainly consisted of quartz fibers, formed in the joint. In addition, the wettability of the AgCuTi on the surface of the quartz fibers was good, and the AgCuTi alloy was promoted by infiltrating into the surface layer of the SiO_2f_/SiO_2_. Furthermore, the “3D-pinning” structure was able to enlarge the joining area between the AgCuTi alloy and the SiO_2f_/SiO_2_ composite, reduce the degree of mismatch of the CTE between the SiO_2f_/SiO_2_ composite and the TC4, and strengthen the shear strength of the joint. However, no good metallurgical bonding formed between the AgCuTi brazing alloy and the fused silica, and the weakening of the E-SiO_2f_/SiO_2_-TC4 joint took place at the bottom of the “3D-pinning” structure. Then, rGO was deposited on the surface of the SiO_2f_/SiO_2_ composite to resolve the difficulty. The microstructure of the rGO@E-SiO_2f_/SiO_2_-TC4 joint is shown in Figure 5c, and it can be found that a sound joint without cracks and voids formed. In addition, the AgCuTi alloy fully reacted with the rGO@E-SiO_2f_/SiO_2_ and Ag(s,s), Cu_3_Ti_3_O, TiO_2_, TiSi_2_ and TiC formed in “3D-pinning-rGO” buffer layer according to XRD pattern of the buffer layer (see in Figure 7). Moreover, the depth of the buffer layer was shallower than that of the SiO_2f_/SiO_2_-TC4 joint. This may have been because the element Ti was consumed by the rGO and formed TiC particles in the buffer layer, which contributed to forming a good transition of the gradient of CTE and strengthened the mechanical property of the rGO@E-SiO_2f_/SiO_2_-TC4 joint. Therefore, it can be inferred that after the selective etching treatment, a “3D-pinning” structure formed in the joint, a good transition gradient of CTE formed and the shear strength of the joint improved. However, a weak joint formed at the bottom of the “3D-pinning” structure due to no metallurgical bonding between the AgCuTi brazing alloy and fused silica. Then, the “3D-pinning-rGO” buffer layer was able to form a good metallurgical bond at the bottom of the structure and strengthened the joint. In addition, the thermal conductivity of the graphene-based materials was excellent, and the presence of the “3D-pinning-rGO” layer facilitated the transfer of heat between the substrates during the brazing process. This may have led to the heat distribution in the rGO@E-SiO_2f_/SiO_2_-TC4 being more efficient and uniform and improving the brazing quality. Therefore, the shear strength of the rGO@E-SiO_2f_/SiO_2_-TC4 reached 43.7 MPa (see Figure 6). The application of the SiO_2f_/SiO_2_ was extended and filled in the blank of the brazing of the SiO_2f_/SiO_2_ with metals.

**Figure 8:**
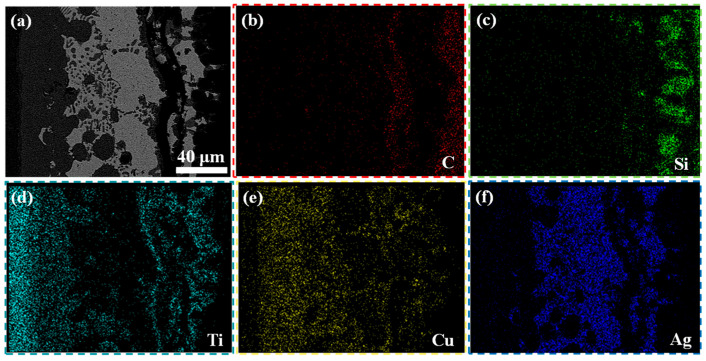
The distribution of the element in rGO@E-SiO_2f_/SiO_2_-TC4 joint (**a**); the microstructure of rGO@E-SiO_2f_/SiO_2_-TC4 joint: (**b**) C, (**c**) Si, (**d**) Ti, (**e**) Cu and (**f**) Ag.

Moreover, the process of element diffusion was able to guide the mechanism of the microstructure of the joint. The distribution of the element in the 2.0 mg/mL rGO@E-SiO_2f_/SiO_2_-TC4 joint is shown in Figure 8. It can be seen that the element Ti mainly diffused from the TC4 and reacted with the element C because the element C coincided with the element Ti beside the SiO_2f_/SiO_2_ composite. In addition, the element Ti also reacted with the element Si because the element Ti was mainly distributed around the quartz fibers. Then, the element Cu and the element Ag were distributed in the brazing seam and no metallurgical bonding occurred. Therefore, it can be inferred that as the brazing process proceeded, the element Ti mainly diffused from the TC4. Then, the element Ti reacted first with the element C and formed the TiC phase. The TiC phase was in the form of particles in the “3D-pinning-rGO” buffer layer, and then the buffer layer was filled by TiC particles. The TiC was in the form of a layer at the bottom of the “3D-pinning-rGO” buffer layer. Therefore, the joining of the AgCuTi alloy and SiO_2f_/SiO_2_ was strengthened by the “3D-pinning-rGO” buffer layer. In addition, the residual element Ti reacted with the element Si, which strengthened the joining of the AgCuTi alloy and quartz fibers.

**Figure 9:**
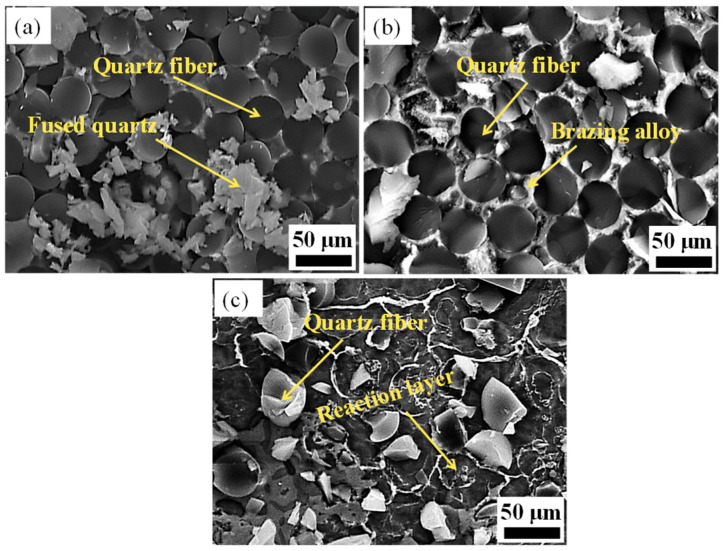
The fracture microstructures of (**a**) SiO_2f_/SiO_2_-TC4 joint, (**b**) E-SiO_2f_/SiO_2_-TC4 joint and (**c**) rGO@E-SiO_2f_/SiO_2_-TC4 joint.

In order to analyze the fracture mechanism of the SiO_2f_/SiO_2_-TC4, E-SiO_2f_/SiO_2_-TC4 and rGO@E-SiO_2f_/SiO_2_-TC4 joints, the fracture surfaces were observed. Figure 8a–c show the fracture surface of the SiO_2f_/SiO_2_-TC4, E-SiO_2f_/SiO_2_-TC4 and rGO@E-SiO_2f_/SiO_2_-TC4, respectively. As shown in Figure 9a, the fracture of the SiO_2f_/SiO_2_-TC4 joint only consisted of broken fibers. This indicates that high residual stress was generated at the brazing seam near SiO_2f_/SiO_2_, and thus, the fracture appeared. As for the fracture microstructure of the E-SiO_2f_/SiO_2_-TC4 joint, broken fibers and the AgCuTi brazing alloy were observed (see Figure 9b). It can be inferred that the fracture developed along the “3D-pinning” structure. In addition, for the fracture of the rGO@E-SiO_2f_/SiO_2_-TC4 joint, broken fibers and alloy products are observed in Figure 9c. This means that the fracture formed in the buffer layer, which corresponds well with the SEM image of the E-SiO_2f_/SiO_2_-TC4 and rGO-E-SiO_2f_/SiO_2_-TC4 joint. According to the above analysis, it can be inferred that the “3D-pinning-rGO” buffer layer could effectively improve the AgCuTi brazing alloy fully infiltrating into the buffer layer and strengthen the reaction between the brazing alloy and the SiO_2f_/SiO_2_. Therefore, the shear strength of the SiO_2f_/SiO_2_-TC4 joint improved from 12.5 MPa to 43.7 MPa (see Figure 6).

### 3.3. Residual Stress Reduction

**Figure 10:**
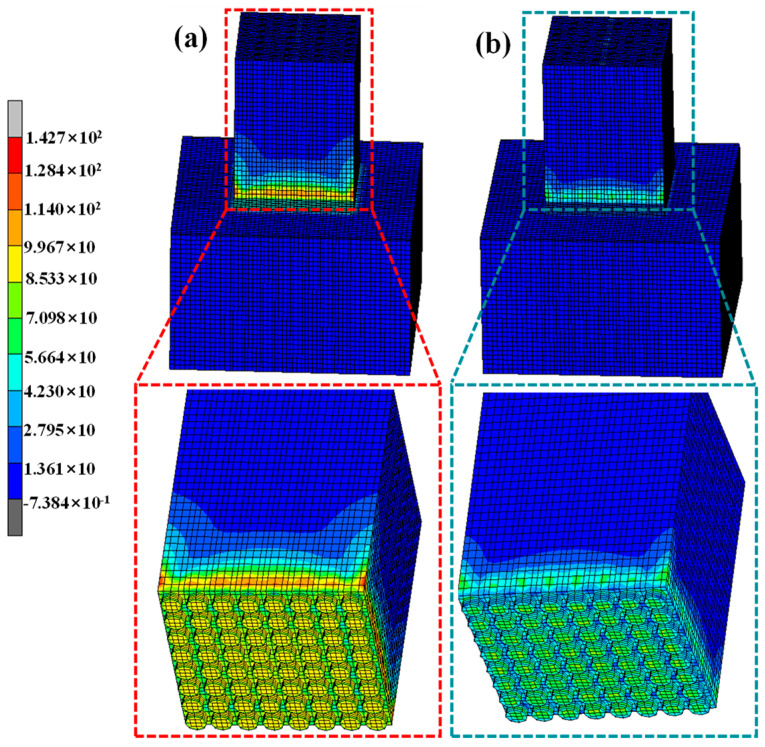
The distribution of the residual stress in (**a**) E-SiO_2f_/SiO_2_-TC4 joint and (**b**) rGO@E-SiO_2f_/SiO_2_-TC4 joint.

Recently, residual stress has received more attention because residual stress plays a vital role in the strength of the joint. And the finite element analysis (FEA) was usually applied to simulate the distribution of residual stress. Using the FEA method, the residual stress in the SiO_2f_/SiO_2_-TC4 brazed joint was analyzed. The model was established according to the analysis of microstructures of the E-SiO_2f_/SiO_2_-TC4 and rGO-E-SiO_2f_/SiO_2_-TC4 joints. The distribution of equivalent von Mises stress in the E-SiO_2f_/SiO_2_-TC4 and rGO-E-SiO_2f_/SiO_2_-TC4 joints are shown in Figure 10 and the distributions of equivalent von Mises stresses in the E-SiO_2f_/SiO_2_ and rGO-E-SiO_2f_/SiO_2_ are shown in the corresponding enlargement area in Figure 10. It can be seen that the residual stress obviously changed after the “3D-pinning-rGO” buffer layer formed. As for the E-SiO_2f_/SiO_2_-TC4 joint, the residual stress was constrained around the interface of the E-SiO_2f_/SiO_2_ and AgCuTi alloy and gradually decreased along the vertical direction of the interface of the E-SiO_2f_/SiO_2_-AgCuTi (see Figure 10a). In addition, the peak residual stress reached about 142 MPa. After the selective etching and depositing treatment, the high residual stress in the rGO@E-SiO_2f_/SiO_2_-TC4 joints transferred from the interface of the E-SiO_2f_/SiO_2_ and the AgCuTi alloy into the “3D-pinning-rGO” buffer layer, as shown in Figure 5b. And the peak residual stress was reduced to 63 MPa. This means that the “3D-pinning-rGO” played an important role in the distribution of the residual stress in the joint. Furthermore, it is worth noting that the maximum residual stress of the rGO@E-SiO_2f_/SiO_2_-TC4 joints transferred from the bottom of the “3D-pinning” structure onto the top of the braided quartz fibers in the “3D-pinning-rGO” buffer layer. In addition, the residual stresses on the E-SiO_2f_/SiO_2_ and braided quartz fibers were reduced with both the etching and depositing treatments, as shown in Figure 10a,b. This may have been because the “3D-pinning-rGO” expanded the area of the SiO_2f_/SiO_2_/AgCuTi, and lowered the mismatch degree of the CTE between the SiO_2f_/SiO_2_ and the TC4. Furthermore, the graphene-based materials had excellent thermal conductivity, and the presence of the 3D-rGO layer facilitated the transfer of heat between the substrates during the brazing process. This led to a more efficient and uniform heat distribution, improving the brazing quality. Therefore, it can be inferred that the “3D-pinning-rGO” buffer layer contributed to expanding the area between the AgCuTi alloy and SiO_2f_/SiO_2_ composite, facilitating the transfer of the heat between substrates, forming a good gradient transition of the CTE, releasing the residual stress in the SiO_2f_/SiO_2_-TC4 joint and improving the shear strength of the joint.

**Figure 11:**
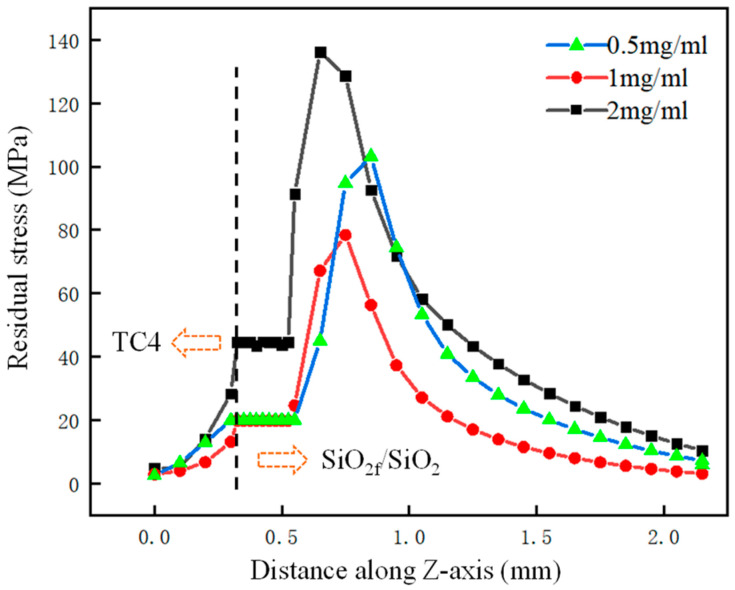
The residual stress with the distance along Z-axis.

According to the above analysis, the rGO contributed to reducing the mismatch degree of the CTE and forming a good gradient transition of the CTE in the SiO_2f_/SiO_2_-TC4 joint. Furthermore, the concentration of the rGO in the “3D-pinning-rGO” buffer layer had an important effect on the residual stress in the rGO-E-SiO_2f_/SiO_2_-TC4 joint. The residual stress with distance along the Z-axis is shown in Figure 11. It can be seen that as the concentration of rGO increased from 0.5 mg/mL to 2.0 mg/mL, the residual stress decreased and then increased. In other words, when the concentration of the rGO was 1.0 mg/mL, the residual stress was the minimum value, which may have been because the mismatching degree of the CTE was minimal. In addition, the residual stress was concentrated in the “3D-pinning-rGO” buffer layer. It is worth noting that the residual stress was concentrated at the bottom of the buffer layer with the 0.5 mg/mL rGO, in the middle of the buffer layer with 1.0 mg/mL and on the top of the buffer layer with 2.0 mg/mL. It can be inferred that as the concentration of rGO increased, the reaction between the AgCuTi alloy and rGO became more intense. Then, the “3D-pinning-rGO” buffer layer was filled with TiC particles and the residual TiC phase was in the form of a layer on the top of the buffer layer. Therefore, the brittle layer transferred from the bottom to the middle and then the top of the buffer layer. 

## 4. Conclusions

In this study, a simple and efficient “3D-rGO” buffer layer was successfully modified on the surface of SiO_2f_/SiO_2_ to assist with brazing with TC4. The effects of the “3D-rGO” buffer layer on the wettability, microstructure and mechanical properties of SiO_2f_/SiO_2_-TC4 are discussed and several conclusions can be summarized as follows:

1. After the selective etching and deposition treatment, the rGO filled the space of fused silica in the surface layer of the E-SiO_2f_/SiO_2_ and a “3D-rGO” buffer layer formed on the surface of the SiO_2f_/SiO_2_. The “3D-rGO” buffer layer contributed to the AgCuTi alloy infiltrating into the surface layer of the SiO_2f_/SiO_2_ and promoting the reaction of the AgCuTi alloy and SiO_2f_/SiO_2_. Wetting experiments showed that after the selective etching treatment, the contact angle reduced from 130° to 55°. When the AgCuTi alloy was on the surface of the rGO@E-SiO_2f_/SiO_2_, the contact angle was further reduced to 38°.
2. A “3D-pinning-rGO” buffer layer formed in the rGO@E-SiO_2f_/SiO_2_-TC4 joint and the typical microstructure was Ag(s,s), Cu_3_Ti_3_O, TiO_2_, TiSi_2_ and TiC. Furthermore, the TiC existed in the form of particles and layers. For the TiC layer, it was able to cause the AgCuTi alloy to infiltrate into the “3D-pinning-rGO” buffer layer and improve the wettability of the AgCuTi alloy on the surface of the SiO_2f_/SiO_2_ composite. And for the TiC particles, they were able to form a good gradient transition of the CTE and release the residual stress of the joint, and thus, the shear strength of the rGO@E-SiO_2f_/SiO_2_-TC4 joint reached 43.7 MPa.
3. The simulation results were consistent with the experiments. The FEM results showed that after the selective etching and deposited treatment, the high residual stress in the rGO@E-SiO_2f_/SiO_2_-TC4 joints transferred from the “3D-pinning” structure in the E-SiO_2f_/SiO_2_ into the “3D-pinning-rGO” buffer layer. Furthermore, for the rGO@E-SiO_2f_/SiO_2_-TC4 joints, the peak residual stress was distributed over the braided quartz fibers in the “3D-pinning-rGO” buffer layer and the peak residual stress reduced from 142 MPa to 63 MPa. In addition, as the concentration of the rGO increased from 0.5 mg/mL to 2.0 mg/mL, the residual stress in the joint transferred from the bottom of the buffer layer to the middle of the buffer layer, and then to the top of the buffer layer.
4. With the combined method of etching and deposited treatment, a “3D-pinning-rGO” buffer layer formed in the rGO@E-SiO_2f_/SiO_2_-TC4 joints. The “3D-pinning-rGO” buffer layer contributed to improving the wettability of the AgCuTi alloy on the surface of the SiO_2f_/SiO_2_. Furthermore, the buffer layer caused released residual stress in the joint, formed a good gradient transition of the CTE and strengthened the shear strength of the joint.

## Data Availability

Data are contained within the article.
